# Impact of national pneumococcal vaccination program on invasive pneumococcal diseases in South Korea

**DOI:** 10.1038/s41598-022-20363-9

**Published:** 2022-09-22

**Authors:** Yeon Haw Jung, Yong June Choe, Chae Young Lee, Sang Oun Jung, Dong Han Lee, Jae Il Yoo

**Affiliations:** 1 Division of Emerging Infectious Disease Response, Korea Disease Control and Prevention Agency, Cheongju, Korea; 2 Department of Pediatrics, Korea University Anam Hospital, 73, Goryeodae-ro, Seongbuk-gu, Seoul, Korea; 3 Division of Emerging Infectious Diseases, Korea Disease Control and Prevention Agency, Cheongju, Korea; 4 Division of Laboratory Diagnosis Analysis, Capital Regional Center for Disease Control and Prevention, Seoul, Korea; 5 Gyeongnam Regional Center, Korea Disease Control and Prevention Agency, Busan, Korea; 6 Division of Bacterial Diseases, Korea Disease Control and Prevention Agency, Cheongju, Korea

**Keywords:** Microbiology, Infectious diseases

## Abstract

Following the introduction of pneumococcal conjugate vaccines (PCVs), the rate of invasive pneumococcal disease (IPD) declined, however, IPDs replaced by serotypes that are not included in the vaccine have emerged. We describe the epidemiology of IPD in South Korea over a 4.5-year period, encompassing the impact following introduction of PCV10/13 and PPSV23 into the public immunization program, and assess serotype dynamics in pediatric and adult population. This was a nationwide, retrospective review of surveillance of all IPD cases in Korea between September 2014 to December 2019. We analyzed VT13 (serotypes included in 13-valent conjugate vaccine) and NVT (nonvaccine type) cases by age, sex, IPD type, vaccination status, and deaths. A total of 893 cases with serotype data were included; 306 (34%) VT13 cases and 587 (66%) NVT cases. Serotype 3 (n = 155) was the most common VT13 serotype, followed by serotypes 19A (n = 70) and 14 (n = 28). Among the NVTs, serotype 10A (n = 74) was the most common serotype, followed by serotypes 23A (n = 60) and 34 (n = 58). Persons who had PCV13 vaccination were at lower risk (aOR = 0.11, 95% CI 0.02–0.73, *P* = 0.022) of death compared to unvaccinated persons. Introduction of PCV10/13 and PPSV23 vaccination program has had different impacts on the serotype-specific IPD across age groups. The most common serotypes included serotypes 3 and 19A (VT13), and 10A, 23A, and 34 (NVT). Our findings suggest continued monitoring in the midst of new vaccine development, and a need to develop novel strategies to mitigate the IPDs from emerging pneumococcal serotypes.

## Introduction

*Streptococcus pneumoniae* is a major cause of morbidity and mortality across all age groups, worldwide^1,2^. Following the introduction of 7-, 10-, and 13-valent pneumococcal conjugate vaccines (PCVs) in childhood population, the rate of invasive pneumococcal disease (IPD) declined, mainly driven by substantial decrease in IPDs caused by serotypes included in the vaccine^3–5^. However, IPDs replaced by serotypes that are not included in the vaccine have emerged in many places of the world, reducing some of the public health benefits from PCVs^6,7^. Changes in distribution of IPD serotypes have not been clearly described on a national scale in Asian countries.

Following the introduction of the PCV7 in Korea in 2003, the proportion of IPD caused by vaccine-type pneumococci has decreased, while non-PCV7 serotypes, especially serotypes 19A and 6A, became predominant among childhood IPD isolates^8^. Before the introduction of 10/13-valent PCV (PCV10/13), pneumococcus accounted for 23.4% of all childhood invasive bacterial diseases in South Korea^9^. While in adults, the estimated annual incidence of IPD ranged from 4.1 to 6.5 cases per 100,000 persons per year, while the case fatality rate was estimated to be 30.8%^10^. The serotypes included in PCV13 accounted for 33.3% of clinical isolates from children and adults^11^. In 2013 and in 2014, South Korea introduced PCV10/13 into the childhood vaccination program and PPSV23 into the elderly vaccination program, respectively^12^. In children, the schedule was to receive the vaccine at 2, 4, 6, and 12–15 months of age; while for adults, once the person has reached 65 years of age^13^. High risk adults were recommended to received PCV13 by academic society, however, was not supported by the national vaccination program^14^. PCV10 and PCV13 were chosen per primary care pediatrician/physician's discretion. There are no out-of-pocket expenses for vaccines included in the NIP in children and adults, and the vaccination coverage rates were 88.3–89.9% in children (2020) and > 60% in adults (2015–2017)^14,15^. It was assumed that PCV13 accounted for more coverage than PCV10, however, the exact rates for the two vaccines have been not reported^15^.

In this study, we describe the epidemiology of IPD in South Korea over a 4.5-year period, encompassing the impact following introduction of PCV10/13 and PPSV23 into a public immunization program, and assess the serotype dynamics in children and adult population.

## Methods

### Setting and study population

This was a nationwide, retrospective review of surveillance of all IPD cases in Korea between September 2014 to December 2019. The population of Korea is around 53 million residents, as of 2022. Our analysis was based on integrated database from the Korea Disease Control and Prevention Agency (KDCA), which collects and merges all reported IPD cases and their vaccination status, in accordance with the Infectious Disease Control and Prevention Act (Article 33-4)^16,17^. IPD was defined as, isolation of *S. pneumoniae* in patients with clinical syndromes from normally sterile sites in the body, such as the blood or cerebrospinal fluid. The national vaccination program provides vaccination guidelines and financial support for vaccines, while the reimbursement of vaccine and service cost is made upon registration of vaccination^18^. The KDCA operates IPD surveillance in accordance with national guideline on vaccine-preventable disease control and prevention, which was adopted and localized from the World Health Organization Surveillance Standards^19^. The surveillance of IPD started in Korea since September of 2014, mandating healthcare providers to report all confirmed or suspected IPD cases to KDCA. It is recommended to submit pneumococcal isolates from normally sterile site to the Division of Bacterial Respiratory Infections, Korea Disease Control and Prevention Agency, Cheongju, Korea. Upon receipt, isolates were sub-cultured using conventional procedures, as described previously^20^, and confirmation and serotyping were conducted through Quellung reaction or multiplex polymerase chain reaction (PCR)^21^. We only included cases that have serotype data. We identified mortality cases (attributable to IPD) through the epidemiologic investigation data.

### Case definition

A confirmed IPD case is defined as positive culture from any normally sterile site (blood, cerebrospinal fluid, pleural fluid, joint fluid) in a symptomatic person. A suspected IPD case in defined as positive polymerase chain reaction (PCR) or antigen test from cerebrospinal fluid. Pneumonia, sinusitis, and otitis media without presence of bloodstream infection are considered non-IPDs, therefore, are not reportable. Epidemiologic investigations in all reported IPD cases are conducted by public health officers.

We defined meningitis as identification of pneumococcus from CSF; and pneumonia as identification of pneumococcus from pleural fluid or bloodstream in cases with clinical diagnosis of pneumonia. All other IPDs (including isolated bacteremia) were grouped as Others.

We classified IPD isolates into 2 groups by serotype: PCV13 serotypes (VT13; serotypes 1, 3, 4, 5, 6A, 6B, 7F, 9V, 14, 18C, 19A, 19F, 23F) and nonvaccine type (NVT; serotypes not included in PCV13). Additional serotypes included in two vaccines under development were sub-grouped as PCV15 additional (serotypes 22F, 33F) and PCV20 additional (8, 10A, 11A, 12F, 15B/C, 22F, 33F); and for serotypes included in the 23-valent pneumococcal polysaccharide vaccine, as PPSV23 additional (8, 9N, 10A, 11A, 12F, 15B/C, 20, 22F, 33F) serotypes.

People receiving two or more doses of 10-valent or 13-valent pneumococcal conjugate vaccinations (PCV10 or PCV13); and one or more doses of 23-valent pneumococcal polysaccharide vaccinations (PPSV23) were considered to be vaccinated.

### Data analysis

We analyzed VT13 and NVT cases by age, sex, IPD type (meningitis, pneumonia, others), vaccination status, and deaths. We compared age-specific incidence per 100,000 population between the baseline period (2015–2017) and 2018–2019, in regard to VT13 and NVT serotypes. We investigated changes in trends in PPSV23 serotypes that are not included in PCV13 in elderly population aged 65+ years. The two-tailed Chi-square test or Student's t test were used to conduct univariate analyses. We conducted multivariate logistic regression to calculate the odds of death by age, types of vaccine (PCV10/13/PPSV23), and number of doses of vaccination. All statistical analyses were conducted using R version 4.0.3.

### Ethical approval

This study was conducted as a legally mandated public health investigation under the authority of the Infectious Diseases Control and Prevention Act (No. 12,444 and No. 13,392) and was exempted by the Institutional Board Review of Korea University Anam Hospital oversight (IRB No. 2021AN0568).

## Results

### IPD cases

Between September 2014 to December 2019, a total of 2,424 IPD cases were reported to KDCA. Of those, 893 cases with serotype data were included in this study; 306 (34%) VT13 cases and 587 (66%) NVT cases (Table 1). Of the VT13 cases, 63.4% (n = 194) were aged 65+ years, whereas 50.6% (n = 297) of NVTs were aged 65+ years (P < 0.001). 5.2% (n = 16) of VT13 cases had meningitis, while 9.9% (n = 58) of NVT cases had meningitis. Of the VT13 cases, 2.3% (n = 7) had vaccinated with PCV13, while 14.3% (n = 84) of NVT cases had PCV13 vaccinated (P < 0.001).

**Table 1:** Characteristics of invasive pneumococcal disease cases by serotype in South Korea, 2014–2019.

Characteristics	VT13^a^		NVT^a^		*P*-value
	No.	%	No.	%	
Total	306		587		
**Age group**					
< 5 years	8	2.6	65	11.1	< 0.001
5–17 years	4	1.3	17	2.9	
18–49 years	22	7.2	64	10.9	
50–64 years	78	25.5	144	24.5	
65+ years	194	63.4	297	50.6	
**Sex**					
Female	98	32.0	200	34.1	0.589
Male	208	68.0	387	65.9	
**Underlying disease**					
Present	177	57.8	337	57.4	0.922
None	128	41.8	247	42.1	
**IPD**					
Meningitis	16	5.2	58	9.9	0.003
Pneumonia	282	92.2	494	84.2	
Other IPD	8	2.6	35	6.0	
**Vaccination status**					
Unvaccinated	80	26.1	121	20.6	< 0.001
PCV10	4	1.3	6	1.0	
PCV13	7	2.3	84	14.3	
PPSV23	117	38.2	175	29.8	
**Outcome**					
Alive	252	82.4	487	83.0	0.924
Death	54	17.6	100	17.0	

*IPD* invasive pneumococcal disease, *PCV10* at least 2 doses vaccination of 10-valent pneumococcal conjugate vaccine, *PCV13* at least 2 doses vaccination of 13-valent pneumococcal conjugate vaccine, *PPSV23* at least 1 dose vaccination of 23-valent pneumococcal polysaccharide vaccine.

^a^VT13, serotypes included in PCV13 (1, 3, 4, 5, 6A, 6B, 7F, 9V, 14, 18C, 19A, 19F, 23F); NVT, serotypes not included in PCV13.

### Serotype by age

**Figure 1:**
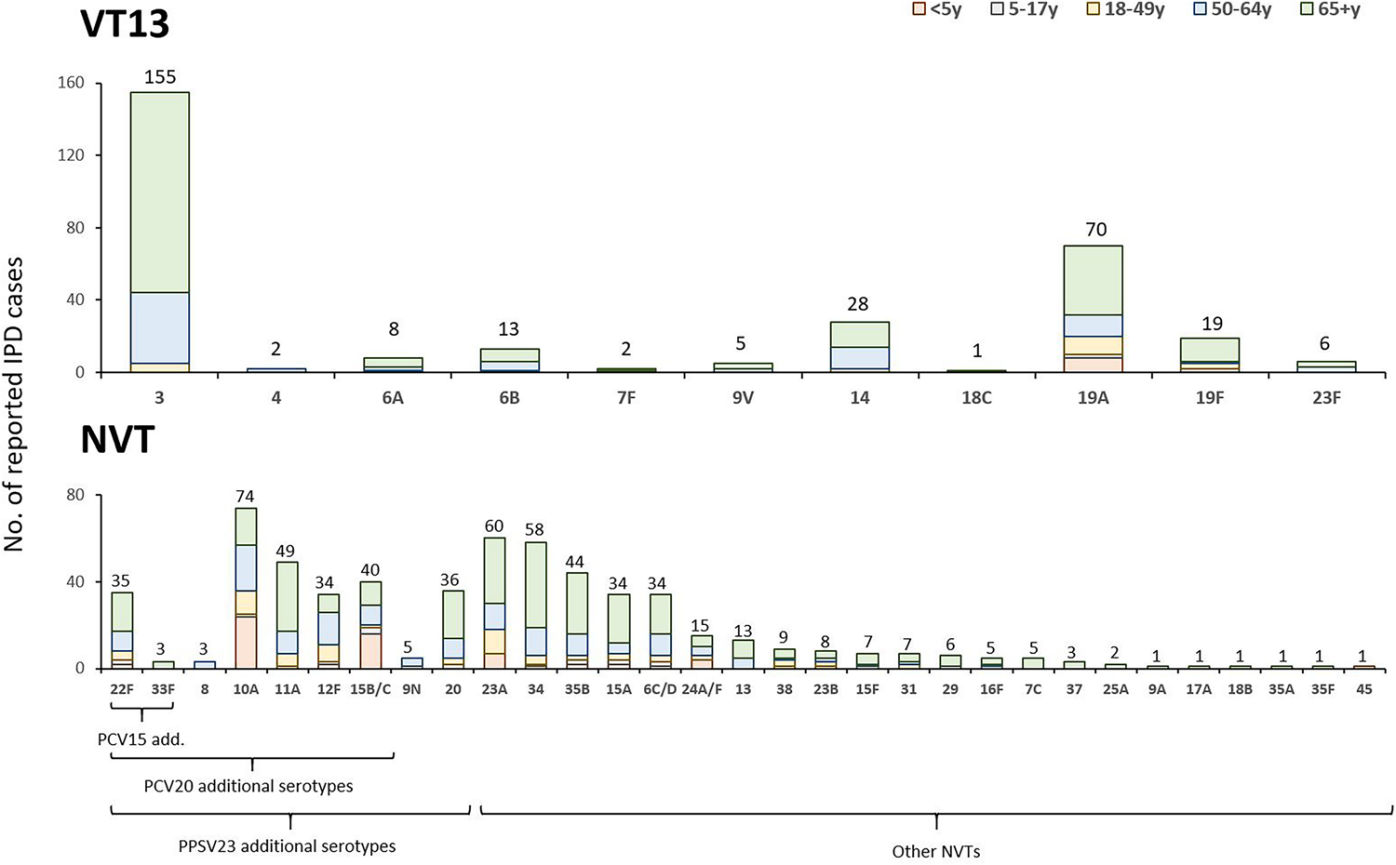
Serotype distribution of pneumococcal isolates form invasive diseases, national surveillance, South Korea, 2014–2019.

In total, serotype 3 (n = 155) was the most common VT13 serotype, followed by serotypes 19A (n = 70), 14 (n = 28), 19F (n = 19), and 6B (n = 13) (Fig. 1). Among the NVTs, serotype 10A (n = 74) was the most common serotype, followed by serotypes 23A (n = 60), 34 (n = 58), 11A (n = 49), 35B (n = 44), and 15B/C (n = 40). Among 587 NVT cases, 6.5% (n = 38) were PCV15 additional serotypes, 40.5% (n = 238) were PCV20 additional serotypes, and 47.5% (n = 279) were PPSV23 additional serotypes.

**Figure 2:**
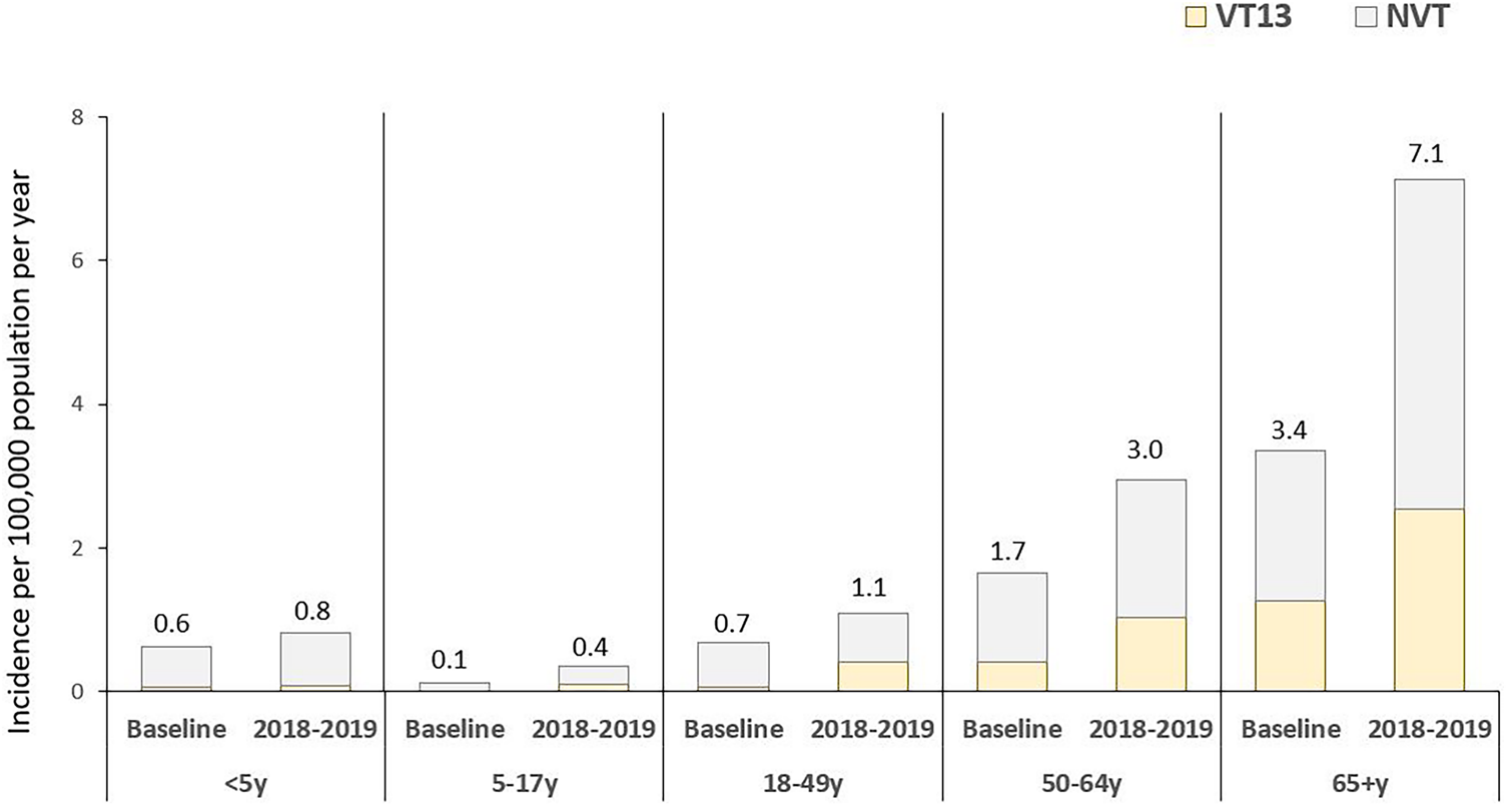
Age-specific incidence per 100,000 population between baseline period (2015–2017) and 2018–2019, in regard to VT13 and NVT serotypes.

The incidence per 100,000 population for VT13 has remained relatively stable in children aged < 5 years and 5–17 years; whereas, the incidence has increased in adults aged 18–49 years (from 0.1 to 0.4 per 100,000), 50–64 years (from 0.4 to 1.0 per 100,000), and 65+ years (from 1.3 to 2.5 per 100,000, Fig. 2). The incidence for NVT has also remained relatively stable in childhood population; however, the incidence has increased in elderly population aged 65+ years (from 2.1 to 4.6 per 100,000). The incidence for total IPD cases for children aged 6-23mo and 24-59mo remained relatively constant (Supplemental Table 1).

**Figure 3:**
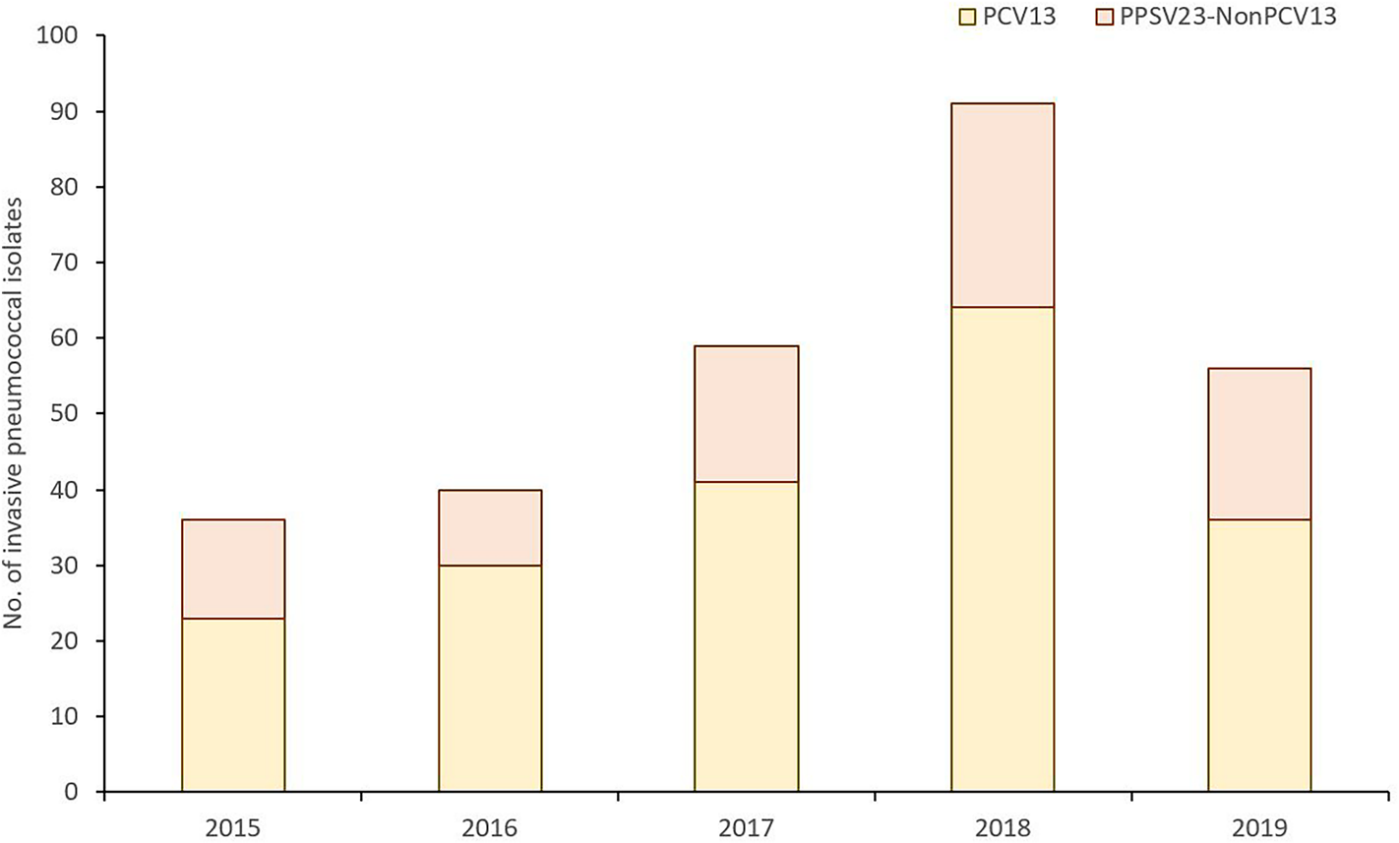
Trend change for PPSV23 serotypes that are not included in PCV13 in elderly population aged 65+ years.

The incidence of PCV13 serotypes in elderly population aged 65+ years ranged from 23 cases in 2015 to 64 cases in 2018 (then, decreased to 36 cases in 2019); while for serotypes that are included in PPSV23, but not in PCV13, the incidence ranged between 13 cases in 2015 and 27 cases in 2018 (Fig. 3).

### IPD death cases

Case fatality rates were 17.6% for VT13 cases and 17.0% for NVT cases (Table 1). After adjusting for variables, persons with pneumonia had higher odds of death compared to meningitis (aOR = 5.93, 95% CI 1.41–24.94, P = 0.015) (Table 2). Persons who had PCV13 vaccinated were at lower risk (aOR = 0.11, 95% CI 0.02–0.73, P = 0.022) compared to unvaccinated persons. After adjusting for variables, serotype 3 had increased risk of death (aOR = 2.32, 95% CI 1.01–5.31, P = 0.046) compared to NVT serotypes (Table 3). Among NVTs, serotypes 9N (aOR = 12.19, 95% CI 1.76–84.31, P = 0.011) and 34 (aOR = 2.90, 95% CI 1.13–7.44, P = 0.027) had increased risk of death compared to VT serotypes.

**Table 2:** Adjusted risk of deaths by characteristics, invasive pneumococcal disease cases, South Korea, 2014–2019.

Characteristics	n/N	%	OR	95% CI	P-value
**Underlying disease**					
Present	97/514	18.9	Ref		
None	57/375	15.2	1.08	0.74–1.58	0.676
**IPD**					
Meningitis	2/74	2.7	Ref		
Pneumonia	146/776	18.8	5.93	1.41–24.94	0.015
**Vaccination status**					
Unvaccinated	56/201	27.9	Ref		
PCV10	0/10	–	–		
PCV13	2/91	2.2	0.11	0.02–0.73	0.022
PPSV23	67/292	22.9	0.76	0.50–1.16	0.205
**Serotype**^a^					
VT13	54/306	17.6	Ref		
NVT	100/587	17.0	1.26	0.86–1.84	0.236

*IPD* invasive pneumococcal disease, *PCV10* at least 2 doses vaccination of 10-valent pneumococcal conjugate vaccine, *PCV13* at least 2 doses vaccination of 13-valent pneumococcal conjugate vaccine, *PPSV23* at least 1 dose vaccination of 23-valent pneumococcal polysaccharide vaccine.

^a^VT13, serotypes included in PCV13 (1, 3, 4, 5, 6A, 6B, 7F, 9V, 14, 18C, 19A, 19F, 23F); NVT, serotypes not included in PCV13.

**Table 3:** Adjusted risk of deaths by serotypes, invasive pneumococcal disease cases, South Korea, 2014–2019.

Serotypes	n/N	%	OR	95% CI	P-value
**VT13**^**a**^					
3	34/153	22.2	2.32	1.01–5.31	0.046
6A	2/8	25.0	2.71	0.47–15.75	0.267
6B	1/13	7.7	0.68	0.08–5.92	0.725
14	3/28	10.7	0.97	0.24–3.97	0.972
19A	10/69	14.5	1.38	0.51–3.72	0.528
19F	4/19	21.1	2.17	0.58–8.15	0.253
**NVT**^**b**^					
22F	4/33	12.1	1.12	0.31–4.02	0.861
11A	14/49	28.6	3.25	1.24–8.50	0.016
12F	1/33	3.0	0.25	0.03–2.12	0.205
15B	4/23	17.4	1.71	0.46–6.31	0.420
9N	3/5	60.0	12.19	1.76–84.31	0.011
20	4/35	11.4	1.05	0.29–3.75	0.942
12F	1/33	3.0	0.25	0.03–2.12	0.205
15A	8/34	23.5	2.50	0.85–7.36	0.097
15B	4/23	17.4	1.71	0.46–6.31	0.420
15F	2/6	33.3	4.06	0.64–25.82	0.137
16F	2/5	40.0	5.42	0.78–37.47	0.087
23A	8/60	13.3	1.25	0.44–3.56	0.676
31	1/7	14.3	1.35	0.14–12.73	0.791
34	15/57	26.3	2.90	1.13–7.44	0.027
35B	12/44	27.3	3.05	1.13–8.20	0.027
6C	7/30	23.3	2.47	0.81–7.58	0.113
7C	2/5	40.0	5.42	0.78–37.47	0.087

^a^VT13, PCV13 vaccine type; in reference to NVT.

^b^NVT, nonvaccine type; in reference to VT13.

## Discussion

Following the introduction of public pneumococcal vaccination programs in South Korea, we observed a differential impact on IPDs between children and adults. Children aged < 5 years accounted for 2.6% of all IPD cases caused by VT13 serotypes, while 11.1% of NVT-induced IPDs were children; whereas 63.4% and 50.6% of VT13-induced and NVT-induced IPDs were adults aged 65+ years, respectively. The finding suggests that in children, more IPDs are occurring from NVT serotypes, while in adults, substantial proportion of IPDs are caused by VT13 serotypes, implicating the need for additional protection. Our finding is in line with reports from North America and Europe, which showed that following the introduction of PCVs, there was emergence of NVT resulting in replacement of serotypes among IPD cases in children^22^. Similarly, in Japan and Taiwan, PCV introduction has resulted in the emergence of NVT replacing VT13 serotypes in IPD cases^23,24^. Despite the higher incidence of NVT-induced IPDs compared to VT13-induced IPDs in Korean children, it is important to note that the overall rate of IPD in the pediatric population is very low, and there is a pronounced protective effect from the PCV13 vaccination. In adults, on the other hand, VT13-induced IPDs remained relatively higher compared to children; likely resulted from lower effectiveness of PPSV23 compared to PCV13. Similarly, in Japan, where PCVs were introduced in childhood population in 2013 and PPSV23 in adult population in 2014, there was only marginal vaccine effectiveness against VT13-induced IPDs in elderly population with 39.2% (95% CI, 2.0–62.2)^25^. After controlling the age group, PCV13 vaccination was significantly associated with lower case fatality rate compared to the unvaccinated group, which was reassuring.

The most common VT13 serotype in children aged < 5 years was serotype 19A, while for adults, serotype 3 was the most common VT13 serotype. The mechanisms for sustained incidence of VT13 serotype may be explained by (1) inadequate vaccination coverage leading to sustained transmission of VT13 serotype; (2) insufficient immunogenicity posed to control the certain serotype; and (3) clonal expansion of pneumococcal lineage associated with antibiotic resistance due to evolutional pressure^26^. In Korea, the increase in serotype 19A was found before the introduction of PCV7, potentially associated with expansion of multidrug-resistant ST320 strain, likely resulted from antibiotic pressure^27^. Serotype 3 has been known to have lower vaccine effectiveness compared to other serotypes^28^. Moreover, the increase of serotype 3 was reported following use of the conjugate vaccines^29,30^. It is concerning that after controlling for demographic and clinical variables, serotype 3 was associated with increased risk of mortality due to IPD. The new PCVs under clinical development should address the limited effectiveness against serotype 3 IPDs.

It is imperative to find the age-specific difference in serotype distribution within NVTs, especially between PCV15 additional serotypes and PCV20 additional serotypes. More than half of serotype 22F (PCV 15-type) were isolated from adult population, whereas nearly 1/3 of serotype 10A and 1/2 of 15B/C (both PCV 20-type) were isolated from children aged less than 5 years of age. Further, the impact of PPSV23 in elderly population was not significant between the observed period. The reason for such difference needs further investigation and monitoring.

This study has number of limitations. First, the surveillance was initiated soon after the PCV10/13 and PPSV23 were introduced to public program, therefore we lack the pre-PCV10/13 data for the children and pre-PPSV23 data for the adult population. We were unable to analyze the secular trend during a short 4.5-year period. Additionally, if reporting of IPD cases increases because of national vaccination program or if identification of cases decreases because of changing clinical practices, then identification of IPD serotypes may not reflect the true burden. Second, cases of bacteremic pneumonia may have been underdiagnosed. However, given the increase in incidence and death from pneumonia cases in South Korea^31^, potentially due to the aging population structure, our finding shows unmet medical needs in severe pneumonia cases that are partly caused by VT13 and NVT serotype pneumococci. Third, serotypes were identifiable in only 1/3 of all reported cases, therefore, the data may not represent the whole population. Moreover, only few portions of IPD cases had serotype data, because the isolates were “recommended”, not “mandated” to conduct serotyping. The serotyping data should be interpreted cautiously because many were excluded from the analysis, indicating changes in surveillance or bias that would affect the analysis. If serotyping had been performed only on severe cases, or if the selection of isolates for serotyping was different between the hospitals, then the observed distribution of serotypes may not reflect the true distribution. Lastly, our finding cannot distinguish between impact from vaccination or from antibiotic pressure. Previous study has delineated the potential influence of penicillin-nonsusceptible genotype on changed serotype distribution in Korea^32^. Future study should incorporate antimicrobial resistance profile of the isolates.

In conclusion, the PCV10/13 and PPSV23 vaccination program have had different impacts on serotype-specific IPD across age groups, potentially through direct and indirect vaccine effects. The most common serotypes included serotypes 3 and 19A (VT13s), and 10A, 23A, and 34 (NVTs). Our finding suggests continued monitoring in the midst of new vaccine development, and the public health need to develop novel strategy to mitigate the IPDs from emerging pneumococcal serotypes.

## Supplementary Information

Supplementary Table 1.

## Data Availability

The data that support the findings of this study are available on request from the corresponding author, YJC. The data are not publicly available due to Personal Information Protection Act of the Republic of Korea.
