# Supplementary material for: Impact of national pneumococcal vaccination program on invasive pneumococcal diseases in South Korea
Source: Sci Rep. 2022 Sep 22;12:15833. doi: 10.1038/s41598-022-20363-9 (PMC9500054; doi:10.1038/s41598-022-20363-9)
Supplement: Supplementary file 1 — Supplementary Table 1. [file 41598_2022_20363_MOESM1_ESM.docx]

**Supplemental Table 1.** Trend change in age-specific incidence of invasive pneumococcal disease per 100,000 population

|  | 2015 | 2016 | 2017 | 2018 | 2019 |
| --- | --- | --- | --- | --- | --- |
| 6-23mo | 1.5 | 3.1 | 4.0 | 3.4 | 3.2 |
| 24-59mo | 1.5 | 2.0 | 1.8 | 1.9 | 1.3 |
